# Narrow *Versus* Standard Diameter Implants for Supporting Single Crown Restorations in the Posterior Jaw: A Randomised Controlled Trial

**DOI:** 10.1016/j.identj.2024.12.031

**Published:** 2025-01-26

**Authors:** Momen A. Atieh, Sunyoung Ma, Andrew Tawse-Smith, Warwick J. Duncan, Fatemeh Amir-Rad, Maanas Shah, Haifa Hannawi, Zaid H. Baqain, Nabeel H.M. Alsabeeha

**Affiliations:** a Mohammed Bin Rashid University of Medicine and Health Sciences, Hamdan Bin Mohammed College of Dental Medicine, Dubai, United Arab Emirates; b Sir John Walsh Research Institute, Faculty of Dentistry, University of Otago, Dunedin, New Zealand; c School of Dentistry, The University of Jordan, Amman, Jordan; d Department of Dental Services, Emirates Health Services, Dubai, United Arab Emirates

**Keywords:** Narrow-diameter dental implants, Patient reported outcome measures, Randomised controlled trial

## Abstract

**Objectives:**

The aim of this randomised controlled trial was to assess clinical, radiographic and patient reported outcomes of narrow *versus* standard diameter titanium zirconium (TiZr) implants supporting single crown restorations in posterior sites with limited bone width.

**Materials and Methods:**

Participants requiring replacement of single missing posterior teeth with implant-supported crowns were randomly allocated into 2 treatment groups: narrow (3.3 mm) or standard (4.1 mm) diameter implant. All implants were restored with screw-retained monolithic zirconia crowns. The changes in marginal bone level (MBL) were assessed at the time of delivery of definitive crown and after 1 year of function. Implant stability was measured at placement, 3 and 12 months. Implant success, pink esthetic score (PES), peri-implant parameters, and patient satisfaction were also evaluated.

**Results:**

A total of 20 participants with 20 implant-supported single crowns completed the 1-year follow-up. All implants were successful. The narrow diameter implants had a higher bone remodeling of 0.39 ± 0.92 mm after 1 year of loading compared with only 0.10 ± 0.29 mm for the standard diameter implants but the difference was not statistically significant (*P* = .40). There were no statistically significant differences between the 2 implant groups in terms of PES and peri-implant outcomes.

**Conclusions:**

Narrow and standard-diameter TiZr implants supporting screw-retained monolithic zirconia crowns in the posterior region were reliable treatment modalities with comparable clinical, radiographic and patient reported outcomes after 1 year of function. Patient satisfaction was high in both treatment groups.

**Clinical relevance:**

The clinical performance of narrow-diameter implants was comparable to standard-diameter implants in replacing a single missing posterior tooth in 12 months. Narrow- and standard-diameter implants can maintain marginal bone levels in 12 months.

## Introduction

Implant supported prostheses is a common treatment modality for the replacement of missing teeth with proven success and predictability. Continued advancements in implant designs and surface modifications over the past decades have aimed to further improve the hard and soft tissue integration of dental implants.^1^ Concurrently, advances in material science and manufacturing techniques have broadened the scope of indications and clinical applications of the implant prosthodontic therapy.^2^^,^^3^ Early implant designs featured standard lengths and diameters and their high survival rates are well-documented.^4^^,^^5^ These conventional implants, however, require sufficient bone volume and adequate interdental distance to prevent adverse bone level changes^6^ or unfavorable long-term esthetic outcomes.^7^ To overcome site specific limitations, simplify treatment protocols, and expand clinical applications, short (<10 mm) and narrow diameter (≤3.5 mm) were introduced.^8^^,^^9^ It has been suggested that narrow-diameter dental implants would allow a less invasive surgical procedure by reducing the need for bone augmentation, a viable option for medically compromised patients and those averse to the use of bone grafting materials.^10^ However the use of narrow diameter implants has been associated with several biological and technical concerns when used over a long period of function.^11^, ^12^, ^13^, ^14^ Therefore, a titanium and zirconium (TiZr) alloy was introduced for fabricating narrow and standard diameter implants with improved mechanical properties. The tensile and fatigue strengths of the TiZr alloys have been shown to be higher than commercially pure titanium (cpTi) and other titanium alloys.^15^

Narrow diameter TiZr implants have demonstrated high survival rates and comparable performance to standard diameter cpTi implants across various clinical indications in several preclinical and clinical studies.^16^, ^17^, ^18^, ^19^ The clinical outcomes of narrow diameter TiZr implants were also comparable to standard diameter implants of the same TiZr material when replacing single missing teeth in the anterior or posterior regions of the maxilla or mandible.^20^^,^^21^ On the other hand, the use of narrow diameter TiZr implants to support single crown restorations exclusively in the posterior region remains limited.^22^^,^^23^ Comparative studies of treatment outcomes using these narrow diameter TiZr implants to standard diameter implants of the same make in the posterior areas remain lacking and evidence from randomised controlled trials on their use is still needed.^8^^,^^24^ The objectives of this single-center randomised controlled trial were to evaluate and compare clinical, radiographic, and patient-reported outcomes of narrow diameter TiZr implants to standard diameter TiZr implants supporting screw-retained monolithic zirconia crowns in posterior sites with limited horizontal bone width.

## Materials and methods

### Patient selection

Patients attending Dubai Dental Hospital between September 2020 and February 2022 and requiring replacement of posterior single missing tooth were invited to take part in the study. Potential participants were screened and selected based on the following inclusion and exclusion criteria.

Inclusion criteria

• Aged 18 or over.
• Controlled oral hygiene (full-mouth plaque and bleeding scores ≤25% at baseline).
• Require replacement of single missing posterior tooth (premolar or molar) with limited bone availability in the horizontal bucco-lingual dimension (residual bone width of ≤6 mm) and a minimum mesio-distal dimension of 7 mm.
• Intact adjacent teeth and opposing dentition.
• Fully healed sites (≥6 months).^25^^,^^26^
• Good compliance and commitment to attend follow-up review appointments.
• Willing to provide informed consent.

Exclusion criteria

• Localised/generalised periodontitis.
• Presence of acute periapical lesion.
• Any medical condition that may contraindicate implant treatment.^27^
• Bone metabolic disease and/or taking medications that affect bone metabolism.
• Long term use of nonsteroidal anti-inflammatory medications.
• History of malignancy, radiotherapy or chemotherapy.
• Pregnant or lactating women.
• Severe bruxism or parafunctional habits.
• Severe occlusal discrepancies.

Participants who smoke ≤10 cigarettes per day^28^ and those with controlled medical conditions and able to undergo surgical procedures under local anesthesia, were not excluded.

The study was approved by the Institutional Review Board of Mohammed Bin Rashid University of Medicine and Health Sciences (MBRU-IRB-2019-20) and registered in the Australian New Zealand Clinical Trials Registry (ACTRN12618001016224). The study was conducted following the ethical standards of the Declaration of Helsinki in 1975, as revised in 2013 and the CONSORT statement was used as a guideline in reporting this study.^29^

### Randomisation, allocation concealment and blinding

The participants were randomly allocated to 2 equal-sized test and control groups (narrow diameter TiZr implant or standard diameter TiZr implant) using computer-generated numbers. Allocation concealment was achieved by selecting and opening sequentially numbered opaque sealed envelopes by a clinician not involved in the study. Group allocation was revealed to the operator just before the surgery. The participants were randomised to one of the following groups:

Test group: Narrow diameter TiZr single implants (diameter 3.3 mm) (Institut Straumann AG)
Control group: Standard diameter TiZr single implants (diameter 4.1 mm) (Institut Straumann AG)

The operator could not be blinded to the intervention due to different diameters of the 2 implants. Blinding of the outcome assessors was only attempted for the assessment of esthetic outcomes as the implant shape and diameter cannot be identified from the clinical photos. Moreover, the statistician was blinded to the allocated group.

#### Treatment procedure and postoperative care

**Fig. 1:**
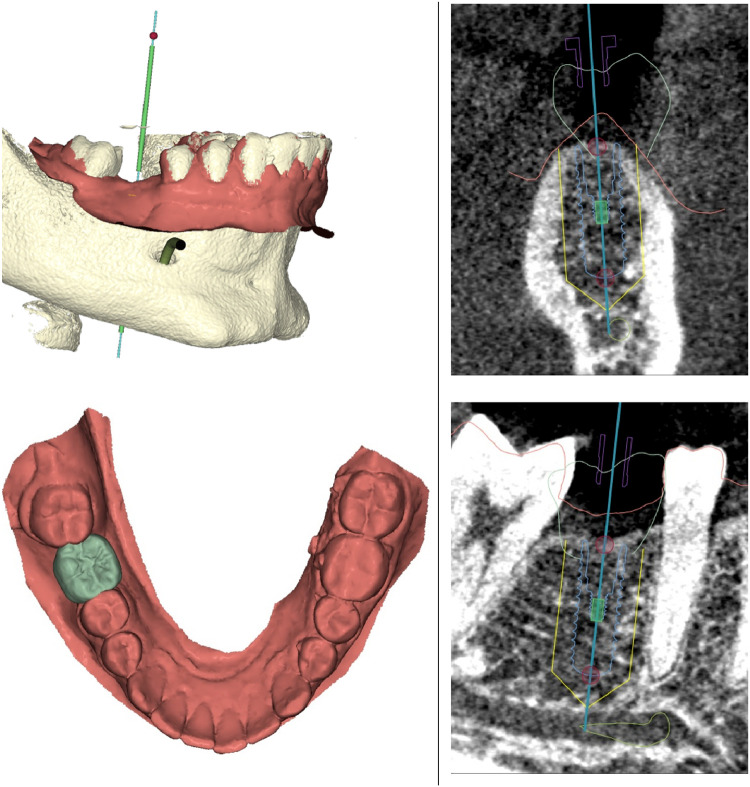
Dental implant planning using a virtual implant software.

Participants underwent comprehensive clinical examination, and radiographic investigations using periapical radiograph and cone beam computed tomography (CBCT) (Orthophos SL 3D, Dentsply Sirona) (Figure 1). Prior to surgery, each participant was asked to rinse with 0.12% chlorhexidine solution (Perio-Aid Intensive Care, Dent-Aid) for 60 seconds. Following the administration of local anesthesia (Lignocaine HCL 2% with 1:100,000 adrenaline) (Novocol pharmaceutical), the implant site was prepared according to the surgical protocol outlined in the ITI consensus conference.^30^ The procedure included raising a mucoperiosteal flap with slight palatal crestal incision and sulcular incision extending to the adjacent teeth. The osteotomy was prepared to receive a chemically modified, sand-blasted and acid-etched TiZr implant of either a narrow (3.3 mm) or a standard (4.1 mm) diameter. In the presence of any peri-implant bone defect, simultaneous contour augmentation with autogenous bone chips, xenograft and resorbable collagen membrane, as described by Buser and co-workers,^31^ was carried out. The flap was sutured with 4/0 polyglactin 910 (Lactisorb Rapid, R1 suture) interrupted and mattress sutures. During the first postoperative week, participants were required to complete a self-reported questionnaire about possible pain, swelling, bleeding, bruising and root sensitivity. The severity of these events was assessed using visual analogue scale (VAS). All participants were instructed to avoid brushing the surgical site for 2 weeks. They were also asked to use anti-inflammatory medication (ibuprofen 600 mg) when needed and 0.12% chlorhexidine mouthwash for 2 weeks. Sutures were removed after 10 to 14 days, and 2-stage protocol was followed with a re-entry procedure after 12 weeks to connect the healing abutment.

**Fig. 2:**
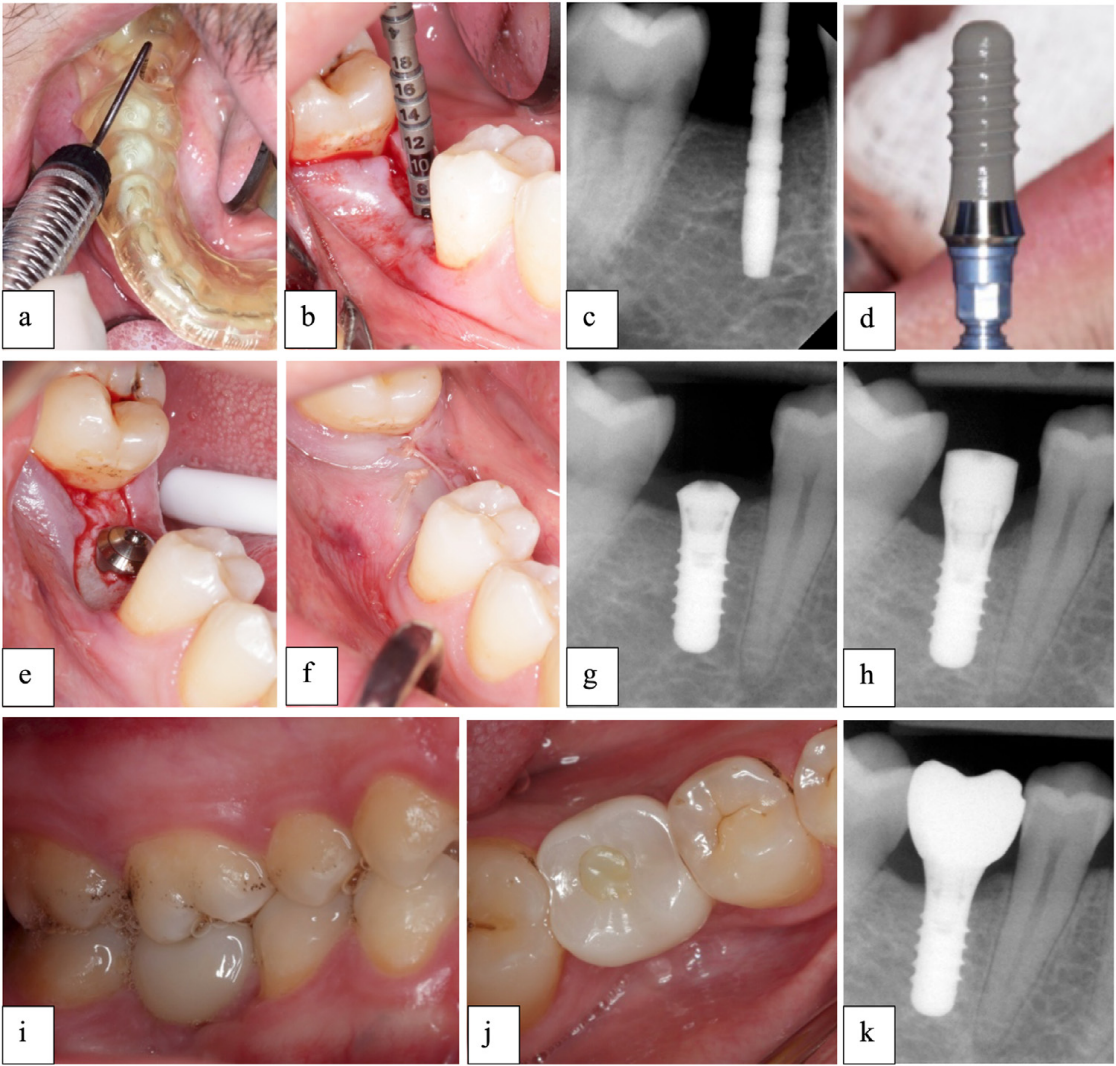
Clinical images of a patient in the standard diameter implant group: (A) surgical guide in situ. (B) osteotomy site with 2.2 mm guiding pin. (C) periapical radiograph of 2.8 mm guiding pin. (D) Straumann Standard Plus Roxolid SLActive RN 4.1 × 10 mm. (E) Dental implant in situ. (F) flap closure with 4-0 PGA resorbable suture. (G) Periapical radiograph immediately after dental implant placement. (H) 3-month post-operative periapical radiograph following placement of healing abutment. (I, J) Delivery of screw-retained implant-supported crown. (K) Peri-apical radiograph immediately after delivery of screw-retained implant-supported crown.

All participants were subjected to similar standardised prosthodontic procedures for the construction of the screw-retained monolithic zirconia implant crowns. A conventional loading protocol was observed^32^ and prosthodontic procedures were commenced 2 to 3 weeks after healing abutment connection. A hybrid analogue digital workflow was followed in the construction of all single crown restorations in accordance with acceptable prosthodontic standards.^22^^,^^33^ Baseline periapical radiographs for marginal bone loss measurements were taken at the conclusion of the crown delivery visit (Figure 2).

### Outcome measures

Primary outcomes:

• Changes in marginal bone level (MBL):

Standardised periapical radiographs were performed at the time of crown delivery and 1 year after loading. Paralleling technique with central rays of the beam on the alveolar crest was applied^34^ and a film holder was used to keep the film parallel to the long axis of the implant and away from the lingual concavity to ensure standardisation of the thread geometry. A computer program (ImageJ, National Institutes of Health) was used to calibrate and measure the digital periapical radiographs.^35^^,^^36^ The known distance between 2 consecutive threads was used to calibrate each radiograph in terms of accounting for any magnification error and calculating the precise pixel to millimeter ratio. The distance between the implant-abutment junction and the first bone-implant contact was measured at the mesial and distal aspect of the implant with the aid of linear measuring toolbar. Average data were obtained for each implant and any bone loss after 1 year of function was presented as a negative value. A single investigator (M.A.) performed all the radiographic measurements. The calibration process was designed to ensure accuracy and consistency in measurements. It included a comprehensive training session in which a total of 5 periapical radiographs were evaluated. These radiographs were measured by the same investigator (M.A.) on separate occasions with a 2-week interval between each measurement. This approach was implemented to assess the intra-examiner reproducibility, ensuring that the investigator's measurements were consistent over time.

Secondary outcomes:

• Implant success/survival rate:

A successful implant was the one that met the criteria proposed by the European Federation of Periodontology.^37^ The criteria included the absence of pain, mobility neuropathy, and periapical radiolucency with no more than 1.5 mm bone loss during the first year of function followed by less than 0.2 mm annually. A survived implant is the one that remained in function but did not meet the success criteria while an implant is considered failed when it was removed.

• Implant stability measurements:

An electronic handheld device (Osstell Beacon, W&H) was used to measure the implant stability at implant placement, 12 weeks after implant placement and 1 year after delivery of definitive crown. The device uses the resonance frequency analysis (RFA) to measure the implant stiffness in implant stability quotient (ISQ) units.^38^^,^^39^ The ISQ scale ranges from 0 to 100 with values higher than 65 ISQ indicating a satisfactory implant stability.^39^, ^40^, ^41^

• Peri-implant parameters:

A manual periodontal probe (UNC-15 periodontal probe, Hu-Friedy) was used to detect bleeding on probing and measure probing pocket depths and recession at 6 points around implants as well as width of keratinised tissue^42^ at 1-year follow-up.

• Esthetic outcomes:

Pink esthetic score (PES) was used to evaluate the peri-implant soft tissues.^43^ The calculation of PES is based on 7 periimplant soft tissue parameters: (1) shape of mesial papilla, (2) shape of distal papilla, (3) level of soft tissue margin, (4) soft tissue contour, (5) deficiency of alveolar process, (6) soft tissue color, and (7) soft tissue texture. Each parameter is scored between 0 and 2 with maximum score of 14. Clinical vestibular and occlusal photographs were taken at the delivery of definitive crown and 1 year after loading. The digital photos were then calibrated based on the clinical length of implant crown and used to calculate PES. Interproximal papillae were assessed for completeness while the other 5 parameters were compared with contralateral tooth. PES of ≥ 8 was considered esthetically acceptable while a PES of ≥ 12 indicated an almost perfect outcome.^44^

• Need for bone augmentation with autogenous bone chips, xenograft and resorbable collagen membrane, as described by Buser and co-workers,^31^ recorded as yes/no at the time of implant placement.
• Patient postoperative experience (pain, swelling, bleeding, bruising, and root sensitivity in adjacent teeth), was evaluated, using a 10 cm VAS from zero (no symptoms) to ten (the worst possible condition), at day 1, 2- and 6-days following implant placement.
• Oral health-related quality of life:

Participants were asked to complete either the English or the Arabic version of the General Oral Health Assessment Index (GOHAI)^45^^,^^46^ prior to implant placement and 1 year after the delivery of definitive crown. GOHAI consists of 12 items to assess participants’ perspectives on oral health, functional and psychological well-being. The 12 items are grouped into 3 categories: (1) Physical function such as chewing and swallowing; (2) Pain or discomfort; (3) Psychological function such as esthetic satisfaction. The overall score ranges between 12 and 60 with each question having 5 possible response items (i.e., constantly/very often, often, sometimes, seldom or never). Participants with high scores were considered to have high satisfaction level.

The safety analysis was performed for all participants. The adverse events were recorded throughout the study according to Organ Class and preferred terms of the Medical Dictionary for Regulatory Activities.^47^

### Sample size calculation

The determination of the sample size needed was based on the changes in MBL between the time of crown delivery and 1 year of function, as per a study by de Souza and colleagues.^48^ It was estimated that a sample size of 9 participants per group was sufficient to detect an effect size of 0.70 and attain a power of 80% with a 2-sided significance level of 5. To achieve the final sample size, it was planned to include a total of 22 eligible participants to compensate for a possible drop-out of 3 participants in each group. The sample size calculation was performed using statistical software (Gpower software, Version 3.1.9.4).^49^

### Statistical analysis

All data (demographic and radiographic measurements) were recorded in Excel spreadsheets and analysed using statistical software (IBM Statistical Package for Social Sciences (SPSS) for windows, Version 28.0. IBM Corp). Descriptive statistics (means, ranges and standard deviations) were used to report study variables. Intra-examiner reliability was examined by intra-class correlation. For continuous parameters such as MBL changes and implant stability measurements, independent t-test was used to compare the 2 treatment groups. Paired t-test was used to identify differences between 2 time points such as the changes in ISQ values between the time of implant placement and subsequent follow-up appointments. When normality assumptions were not satisfied, the equivalent non-parametric tests were applied. For categorical parameters such as implant success rate, chi-square test was employed. *P*-value less than .05 indicated statistical significance. The unit of analysis was the participant.

## Results

A total of 22 participants met the inclusion criteria, signed the informed consent and received single implants in the posterior region. Only 20 participants completed the one-year observation period as 2 participants missed the scheduled one-year follow-up appointments and could not be reached (Figure S1). There were more standard diameter TiZr implants placed in molar sites compared with narrow diameter implant group (61.5% *versus* 38.5% of molar sites) but the difference was not statistically significant. Both treatment groups were comparable with respect to demographics and site characteristics (Table S1).

### Surgical outcomes

At the time of implant placement, 1 standard and 4 narrow diameter implants required bone augmentation. None of the implants failed at the end of the first year of function. However, 2 implants in the narrow diameter group were lost to follow-up following the placement of definitive crown despite constant effort to reach the participants. All the 20 implants evaluated at the one-year follow-up visit met the success criteria, thus achieving 100% success and survival rates.

#### Radiographic analyses

**Fig. 3:**
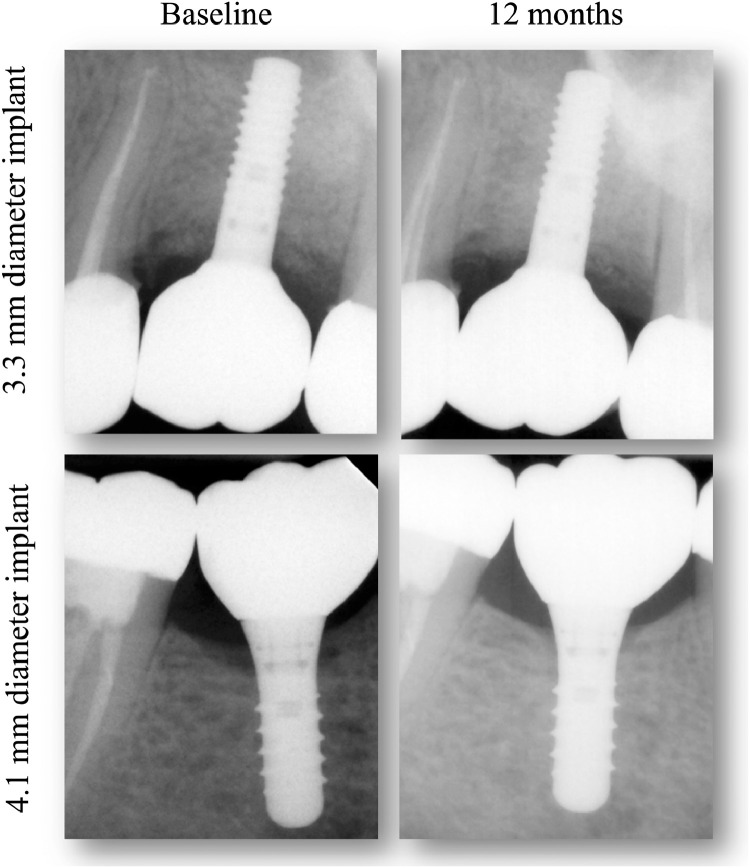
Radiographic assessment of marginal bone levels around narrow and standard diameter implants supporting single screw-retained monolithic zirconia crowns

The intraclass correlation coefficient ranged between 0.77 and 0.98 for radiographic measurements, indicating substantial to almost perfect intra-examiner agreement. The baseline means of MBL at the time of crown delivery in the narrow diameter and standard diameter TiZr implant groups were 2.12 ± 1.09 mm, and 1.48 ± 0.56 mm, respectively, while means of MBL at 1 year after loading in the narrow diameter and standard diameter TiZr implant groups were 1.74 ± 0.78 mm, and 1.38 ± 0.43 mm, respectively. In both treatment groups, the mean changes in MBL over 1 year of function were not significant. However, the narrow diameter TiZr implants had a higher bone remodeling of 0.39 ± 0.92 mm after 1 year of loading compared with only 0.10 ± 0.29 mm for standard diameter TiZr implants. Still, the difference was not statistically significant (*P* = .40) (Table 1, Figure 3).

**Table 1:** Radiographic peri-implant marginal bone level changes.

Marginal bone changes from baseline to 1-year follow-up				
	Mean (SD)	Mean difference (SD)	95% CI	*P*-value
Narrow diameter TiZr implant group*				
Baseline	2.12 (1.09)	0.39 (0.92)	−0.32, 1.09	.24
One year	1.74 (0.78)			
Standard diameter TiZr implant group*				
Baseline	1.48 (0.56)	0.10 (0.29)	−0.09, 0.30	.27
One year	1.38 (0.43)			


Differences in marginal bone level changes between the 2 groups after 1 year				
Narrow diameter TiZr implant group† mean (SD)		Standard diameter TiZr implant group† mean (SD)	Mean difference and 95% CI	*P*-value
0.39 (0.92)		0.10 (0.29)	−0.28 (−0.90, 0.33)	.40

TiZr, titanium-zirconium; CI, confidence interval; SD, standard deviation.

⁎ Paired *t*-test.

† Independent *t*-test.

#### Implant stability

Standard diameter TiZr implants showed a higher mean ISQ value than narrow diameter TiZr implants at the time of implant placement (75.73 ± 5.15 *versus* 75.09 ± 6.17 ISQ units) but the difference was not significant (*P* = .80). In contrast, the difference between the 2 implant groups was statistically significant at the second-stage implant surgery at 12 weeks (*P* = .003), with higher mean ISQ value for standard diameter TiZr implants compared with narrow diameter TiZr implants (81.05 ± 2.29 *versus* 75.85 ± 4.53 ISQ units). At 1 year of function, the standard diameter TiZr implants maintained higher mean ISQ values compared with narrow diameter TiZr implants (84.68 ± 3.30 *versus* 81.80 ± 2.71 ISQ units) but without any statistically significant difference (*P* = .11). The effects of patient and site-related factors on implant stability measurements at 3-time points are summarised in Table 2.

**Table 2:** Changes in implant stability measurements by patient- and site-related factors.

ISQ values at implant placement			
Variable	Mean (SD)	Mean difference and 95% CI	*P*-value

Gender			
Female	73.55 (6.47)	−3.41 (−8.24, 1.42)	.16
Male	76.96 (4.36)		
Age (years)			
26-50	75.09 (5.06)	−1.16 (−6.82, 4.50)	.68
51-75	76.25 (7.20)		
ASA I	75.44 (5.16)	0.14 (−5.90, 6.18)	.96
ASA II	75.30 (7.45)		
Smoking habits			
Non-smokers	75.45 (5.84)	0.45 (−8.36, 9.26)	.92
Smokers	75.00 (0.00)		
Bone quality*			
Type II	76.83 (4.52)	3.13 (−1.74, 8.00)	.20
Type III	73.70 (6.41)		
Implant length (mm)			
8-10	76.21 (4.55)	3.51 (−6.52, 13.54)	.41
12	72.70 (8.22)		
Implant diameter (mm)			
3.3	75.09 (6.17)	−0.64 (−5.69, 4.42)	.80
4.1	75.73 (5.15)		
Need for augmentation at time of implant placement			
Yes	73.10 (8.54)	2.99 (−7.44, 13.42)	.49
No	76.09 (4.49)		

ISQ values at second stage implant surgery			
Variable	Mean (SD)	Mean difference and 95% CI	*P*-value

Gender			
Female	78.56 (3.16)	−0.03 (−4.15, 4.09)	.99
Male	78.58 (5.21)		
Age (years)			
26-50	77.93 (4.89)	−2.23 (−6.62, 2.15)	.30
51-75	80.17 (2.14)		
ASA I	78.00 (4.64)	−2.40 (−7.05, 2.25)	.29
ASA II	80.40 (2.88)		
Smoking habits			
Non-smokers	78.37 (4.38)	−2.13 (−9.00, 4.74)	.52
Smokers	80.50 (4.95)		
Bone quality*			
Type II	78.13 (4.35)	−1.04 (−5.13, 3.05)	.60
Type III	79.17 (4.54)		
Implant length (mm)			
8-10	78.69 (3.71)	0.49 (−4.29, 5.27)	.83
12	78.20 (6.53)		
Implant diameter (mm)			
3.3	75.85 (4.53)	−5.20 (−8.43, −1.96)	.003
4.1	81.05 (2.29)		
Need for augmentation at time of implant placement			
Yes	74.40 (6.02)	5.48 (−1.90, 12.85)	.11
No	79.88 (2.82)		

ISQ values after 1 year of loading			
Variable	Mean (SD)	Mean difference and 95% CI	*P*-value

Gender			
Female	84.17 (1.97)	0.62 (−3.19, 4.43)	.73
Male	83.55 (4.03)		
Age (years)			
26-50	83.55 (4.21)	−0.62 (−4.43, 3.19)	.73
51-75	84.17 (1.08)		
ASA I	82.83 (2.25)	−3.79 (−11.10, 3.51)	.21
ASA II	86.63 (4.77)		
Smoking habits			
Non-smokers	83.90 (3.42)	1.90 (−5.67, 9.47)	.60
Smokers	82.00 (0.00)		
Bone quality*			
Type II	84.22 (4.37)	1.01 (−2.68, 4.70)	.57
Type III	83.21 (1.32)		
Implant length (mm)			
8-10	84.41 (3.60)	2.01 (−1.82, 5.83)	.28
12	82.40 (2.41)		
Implant diameter (mm)			
3.3	81.80 (2.71)	−2.88 (−6.52, 0.76)	.11
4.1	84.68 (3.30)		
Need for augmentation at time of implant placement			
Yes	83.00 (3.19)	1.04 (−3.19, 5.28)	.61
No	84.04 (3.48)		

ASA, American Society of Anesthesiologists; ISQ, implant stability quotient; CI, confidence interval; SD, standard deviation.

⁎ According to Lekholm and Zarb (1985). Chi-square test.

#### Peri-implant parameters

The number and percentage of implants with presence of bleeding on probing, probing pocket depths of > 5 mm and width of keratinised tissue of less than 2 mm are summarised in Table S2. At 1 year of functional loading, 3 implants presented with bleeding on probing on at least one site, while one of the implants had probing pocket depths of more than 5 mm at one site.

#### Esthetic outcomes

The PES ranged between 7 and 14 with no statistically significant difference between narrow and standard diameter TiZr implants at the time of crown delivery (9.82 ± 2.23 *versus* 11.55 ± 2.16; *P* = .08) or after 1-year of functional loading (8.67 ± 1.50 *versus* 10.64 ± 2.46; *P* = .05). Individual comparisons between the 2 implant groups for mesial papilla, distal papilla, soft tissue margin, tissue contour, alveolar process, colour and texture did not show any statistically significant difference (Table S3).

### Patient-reported outcome measures

All the participants completed the VAS following implant placement. The mean pain, swelling, and bleeding VAS scores ranged between 2 and 4 in the first 2 days following implant placement and then reduced to less than 2 in the 6th postoperative day. No statistically significant differences between narrow and standard diameter implants were detected for pain, swelling, bleeding, bruising and root sensitivity at any time point (Table S4). Apart from those stated in the questionnaire, no other adverse events were reported.

The completion rates of the GOHAI prior to implant surgery and 1 year after functional implant loading were 100% and 88.9%, respectively. At baseline, the mean GOHAI scores were 47.91 ± 2.98 and 47.91 ± 2.07 for narrow and standard diameter TiZr implants, respectively, indicating a relatively inadequate oral health-related quality of life (i.e., < 50).^46^ The mean GOHAI scores across all domains improved after delivering the definitive crowns, with total GOHAI scores of 53.89 ± 2.42 for narrow diameter TiZr implants and 53.18 ± 1.78 for standard diameter TiZr implants (Table 3).

**Table 3:** Changes in GOHAI scores from baseline to one-year follow-up.

GOHAI domain	Mean (SD)	Mean difference (SD)	95% CI	*P*-value
Narrow diameter TiZr implant group*				
Physical function				
Baseline	16.44 (1.24)	−1.22 (1.30)	−2.22, −0.22	.02
One year	17.67 (1.00)			
Pain/discomfort				
Baseline	11.89 (1.17)	−1.89 (0.78)	−2.49, −1.29	<.001
One year	13.78 (0.83)			
Psychological function				
Baseline	19.67 (1.58)	−2.78 (1.20)	−3.70, −1.85	<.001
One year	22.44 (1.13)			
**Total score**				
Baseline	48.00 (3.24)	−5.89 (2.03)	−7.45, −4.33	<.001
One year	53.89 (0.81)			
Standard diameter TiZr implant group*				
Physical function				
Baseline	16.45 (0.31)	−0.91 (1.04)	−1.61, −0.21	.02
One year	17.36 (0.20)			
Pain/discomfort				
Baseline	11.45 (0.69)	−2.18 (0.98)	−2.84, −1.52	<.001
One year	13.64 (1.03)			
Psychological function				
Baseline	20.09 (1.30)	−2.00 (1.00)	−2.67, −1.33	<.001
One year	22.09 (0.83)			
**Total score**				
Baseline	47.91 (2.07)	−5.18 (2.04)	−6.55, −3.81	<.001
One year	53.09 (1.87)			

GOHAI, general oral health assessment index; TiZr, titanium-zirconium; CI, confidence interval; SD, standard deviation

⁎ Paired *t*-test

The safety record did not show any adverse events related to the oral soft and hard tissues in either implant group. In the narrow-diameter implant group, one participant experienced an upper respiratory tract infection within the 2 weeks following the implant surgery, but that event was not related to the intervention.

## Discussion

This single-center study with one-year follow-up compared clinical, radiographic and patient-reported outcomes for narrow (3.3 mm) diameter or standard (4.1 mm) diameter TiZr implants supporting screw-retained monolithic zirconia crowns in posterior ridges with limited bone width. There were no significant differences between the 2 implant groups in radiographic changes in MBL, implant stability, peri-implant parameters, or patient-reported outcomes up to 12 months after delivery of definitive crowns. The narrow-diameter implants had a slightly more bone remodeling and higher GOHAI score than the standard-diameter implants while standard-diameter implants had a higher PES after 12 months of function.

A number of systematic reviews^8^^,^^14^^,^^24^^,^^50^, ^51^, ^52^, ^53^ have evaluated the use of narrow diameter implants made of TiZr or different Ti alloys in anterior and posterior sites in terms of implant survival and changes in MBL. Overall, narrow implants of 3.3 mm diameter had high survival rates ranging between 93% to 100% over a period up to 3 years.^50^ The survival rates of narrow TiZr implants were also comparable to standard diameter implants or narrow diameter implants made of commercially pure Ti alloy.^14^^,^^51^^,^^52^ The mean differences in MBL ranged between 0.36 ± 0.06 mm^51^ to 0.01 ± 0.65^52^ after 1 year of function with no significant differences between narrow and standard diameter implants. These systematic reviews identified only one study^54^ where changes in MBL were significantly different and in favor of standard diameter implants. The findings of the present study in terms of success/survival rates were in accordance with the overall survival rates reported in the above-mentioned systematic reviews. Our mean difference in MBL resembled the systematic review by Altuna and co-workers^51^ but differed from the retrospective study by Zweers and co-workers.^54^ The latter showed that bone loss around narrow diameter implants supporting overdentures was significantly higher than standard diameter implants. This could be related to the longer observation period of the retrospective study (i.e., 3-year *versus* 1-year follow-up) and the use of narrow diameter implants of different surface characteristics in fully edentulous scenarios. Moreover, the stress distribution around narrow 3.3 mm diameter Ti implants supporting overdentures could be different where van Mises stress and strain values are likely to increase to levels that might induce secondary bone resorption.^55^, ^56^, ^57^

It is worth noting that the systematic reviews of narrow diameter implants included both single sites and partially edentulous cases, and both anterior and posterior sites; it was not possible to individually report on success rates or changes in MBL in posterior sites. Moreover, none of the systematic reviews have quantitatively reported on implant stability, peri-implant parameters, esthetic outcomes or patient reported outcomes to allow comparison with the present findings. However, one randomised controlled trial^22^^,^^58^ has specifically reported on the clinical outcomes of single narrow (3.3 mm) diameter TiZr implants in molar regions. This showed failure rates of 1% and 18% at one and 3-year follow-up, respectively. The failed narrow diameter implants were regular neck (RN) with a prosthetic platform of 4.8 mm and were mostly lost without any signs of inflammation after the first year of function. While none of the narrow diameter implants in the present study failed, there has only been 1 year of follow-up to date and all were narrow neck crossfit (NNC) with a prosthetic platform of 3.5 mm. It is still premature to make any comparison between the 2 studies, but one could speculate that the implant design might have contributed to the high failure rate over time. The prosthetic platform of 4.8 mm in diameter might have caused more leverage on a narrow diameter RN implant body placed in a site with high occlusal load (i.e., single molars) compared to the 3.5 mm prosthetic platform of the NNC implants used in this study.

The clinical use of the narrow diameter NNC implants warrants careful consideration, particularly in the context of its historical development and primary indications. Specifically, this implant was designed when other implants, such as bone level implants were not yet available and was primarily indicated for the replacement of incisors.^8^ The historical context and specific design intentions of the narrow diameter NNC implants must be taken into account when interpreting and assessing the external validity of our results. One of the anticipated benefits of using narrow diameter implants is the ability to overcome anatomical challenges and replace missing teeth in atrophic ridges without the need for bone augmentation.^14^^,^^51^ However, the present study failed to demonstrate the advantageous use of narrow diameter implants in this perspective since 4 out of the 5 cases that required simultaneous contour augmentation belonged to the narrow diameter implant group. This could be attributed to 2 factors; firstly, the inclusion of posterior sites where sufficient bone width might have been present even when it is considered limited (≤ 6 mm) as per the inclusion criteria and secondly, the number of cases was not enough to allow reliable comparison between the 2 implant groups in this perspective.

High levels of implant stability were recorded at implant placement and after 3 and 12 months without any significant difference between both implant groups, except at the 3-month follow-up where standard diameter implants had significantly higher ISQ values than narrow diameter implants. Previous studies^59^^,^^60^ have demonstrated that implant diameter was not a significant factor in improving implant stability up to 3 months following implant placement. It is worth noting that contradictory results have been reported in the literature,^36^ as one cohort study^21^ showed that 4.1 mm diameter implants had significantly higher implant stability values than 3.3 mm diameter implants after 1 year of function. The authors attributed the significant difference to the greater surface area of 4.1 mm diameter implants and hence larger contact area for osseointegration. Implant stability could also be influenced by other factors such as bone morphology and implant length.^61^^,^^62^ In this context, the recorded ISQ values in the present study were not influenced by patient-related factors such as age and gender, site-related factors such as bone quality, or other implant-related factors such as implant length at any time point. It could be speculated that none of these factors, including implant diameter, had any significant effect on the level of implant stability once it reaches certain level, or the sensitivity of the RFA device might not have been enough to detect small differences between the 2 implant groups (3.3 mm *versus* 4.1 mm diameter implants).

Esthetically acceptable outcomes were recorded for both implant groups with the standard diameter implants having slightly higher PES than narrow diameter implants at one-year follow-up. It should be noted that esthetic outcomes of implant prostheses are mostly influenced by anatomical factors, periodontal biotype, and implant position^7^ rather than merely an implant diameter. In this context, all the implants except for one in the narrow diameter implant group had a width of keratinised tissue of more than 2 mm indicating a favorable periodontal biotype. All implants were also placed in a restoratively driven fashion respecting the apico-coronal and mesio-distal dimensions to ensure optimum esthetic and biomechanical results. Moreover, the recorded mean PES in the present study was similar to average scores in studies reporting esthetic outcomes of narrow and standard diameter implant-supported prostheses in the anterior maxilla.^21^^,^^63^

In terms of peri-implant variables, a split-mouth randomised controlled trial,^48^ including twenty patients with both narrow 3.3 mm implants and standard 4.1 mm implants, reported on peri-implant parameters after one and 3 years of function. Four out of 20 implants presented with bleeding on probing while only one implant presented probing pocket depths of > 5 mm after 1 year of function. Our study had similar outcomes as the number of implants presenting with bleeding on probing were 2 out of the 16 implants that completed the one-year follow-up while none of the implants presented with probing pocket depths of > 5 mm.

The participants’ perception of pain, swelling, bleeding, bruising and root sensitivity during the first week following implant placement was evaluated using VAS. The scores were in the range of 2 to 4 in the first 2 days and then decreased to less than 2 on the 6th postoperative day indicating a well-tolerated surgical procedure without any significant difference between the 2 implant groups. The VAS scores were similar to one study^64^ that evaluated the factors influencing postoperative pain. The authors concluded that the postoperative pain intensity is not affected by implant diameter. The additional drilling required to place standard diameter implants compared to narrow diameter implants had clearly no impact on the postoperative experience. An explanation for the low scores could also be related to the surgical procedure being performed in a university clinic setting by experienced periodontists^65^ and the exclusion of patients who were heavy smokers or had uncontrolled medical conditions. Moreover, simultaneous contour augmentation was not required in the majority of the cases which might have resulted in a more straightforward surgical procedure.

High patient satisfaction in the 3 domains of GOHAI was noticed in both groups with means scores above 50 after 1 year of function. Similar comparative data is limited as only 2 studies,^54^^,^^66^ evaluating narrow and standard diameter implants, reported on patient reported outcomes and used different instruments. In one study,^66^ patients were asked to assess the esthetic and function of their implant restorations by completing a VAS after more than 3 years of function. The mean values for esthetics and function were 88% and 96%, respectively suggesting highly satisfied participants irrespective of the implant diameter. Another study^54^ used a scale (0-10) to assess patient satisfaction after 1 and 3 years of function. Although the patient satisfaction scores were significantly higher for the narrow diameter implants and locator attachment group compared to standard diameter implants and ball attachment group at the first year of function, both implant groups scored above 8 with no statistically significant difference after 3 years.

The placement of narrow diameter implants in the posterior jaw, particularly in molar sites, can lead to the fabrication of implant crowns with restorative emergence angles and profiles that may not allow optimal interproximal plaque removal and increase the risk of peri-implant diseases. Several studies have shown that prosthetic design features like implant-abutment connection and retention mechanism are associated with a high incidence of peri-implant mucositis and peri-implantitis.^67^^,^^68^ However, the impact of the implant restorative emergence angle and/or profile on changes in soft and hard tissues surrounding implants has received little attention. The effect of these prosthetic elements on the marginal bone level and incidence of peri-implant diseases has been the subject of recent investigations with inconsistent findings.^69^, ^70^, ^71^, ^72^, ^73^, ^74^ A recently published systematic review^75^ indicated that implant restorations with an emergence angle of 30 degrees or less were not superior to those with an angle greater than 30 degrees in terms of changes in marginal bone levels. However, the review provided moderate to low certainty evidence suggesting that platform-matched implant restorations with an emergence angle of 30 degrees or less might be associated with fewer changes in marginal bone levels compared to those with an emergence angle greater than 30 degrees. In our future follow-ups, we will investigate the impact of prosthetic design on peri-implant health to better understand its role in the changes in marginal bone level, onset and progression of peri-implant diseases.

The current study attempted to report the essential outcomes and measurements as recommended by a recently published consensus paper.^76^ Nevertheless, this report has limitations which include a short observation period and small sample size. The sample size was calculated to specifically compare the changes in MBL without taking into account other outcomes, and hence there is a potential for underpowered analysis. The radiographic findings, however, were relatively consistent with other studies^19^^,^^77^ and a systematic review.^51^ Additionally, our findings might have been influenced by other potential confounding factors like implant length and bone quality. In this context, we have attempted to control for demographic and site characteristics. The range of implant lengths used in this study does not have any influence on implant stability^78^ nor the encountered Type II/III bone quality have shown to have any significant impact on the long-term marginal bone level changes.^79^ None of the implant sites were estimated to have Type IV bone quality, a quality which is often associated with more loss of marginal bone^80^ and could have been considered as a potential confounding factor.

## Conclusions

Within the limitation of this study, narrow and standard diameter implants supporting single screw-retained monolithic zirconia crowns in the posterior region were reliable treatment modalities with comparable and favorable changes in MBL, implant stability and esthetic outcomes after 1 year of function. High patient satisfaction was achievable in both groups.

## Data availability statement

The data that support the findings of this study are available on the request from the corresponding author. The data are not publicly available due to privacy and ethical approval.

## Author contributions

Momen A. Atieh: Concept/design, data collection, data analysis/interpretation, drafting article, critical revision of article, approval of article. Sunyoung Ma: Critical revision of article, approval of article. Andrew Tawse-Smith: Critical revision of article, approval of article. Warwick Duncan: Critical revision of article, approval of article. Fatemeh Amir-Rad: Data collection, critical revision of article, approval of article. Maanas Shah: Data collection, critical revision of article, approval of article. Haifa Hannawi: Critical revision of article, approval of article. Zaid H. Baqain: Critical revision of article, approval of article. Nabeel H. M. Alsabeeha: Data collection, data analysis/interpretation, drafting article, critical revision of article, approval of article.

## Conflict of interest

None disclosed.

## Appendix Supplementary materials

Image, application 1

Image, application 2

Image, application 3

## Footnotes

Supplementary material associated with this article can be found in the online version at doi:10.1016/j.identj.2024.12.031.
