# Supplementary figures and images for: Narrow Versus Standard Diameter Implants for Supporting Single Crown Restorations in the Posterior Jaw: A Randomised Controlled Trial
Source: Int Dent J. 2025 Jan 26;75(3):2071–83. doi: 10.1016/j.identj.2024.12.031 (PMC12142787; doi:10.1016/j.identj.2024.12.031)

**Figure S1:** CONSORT flow diagram

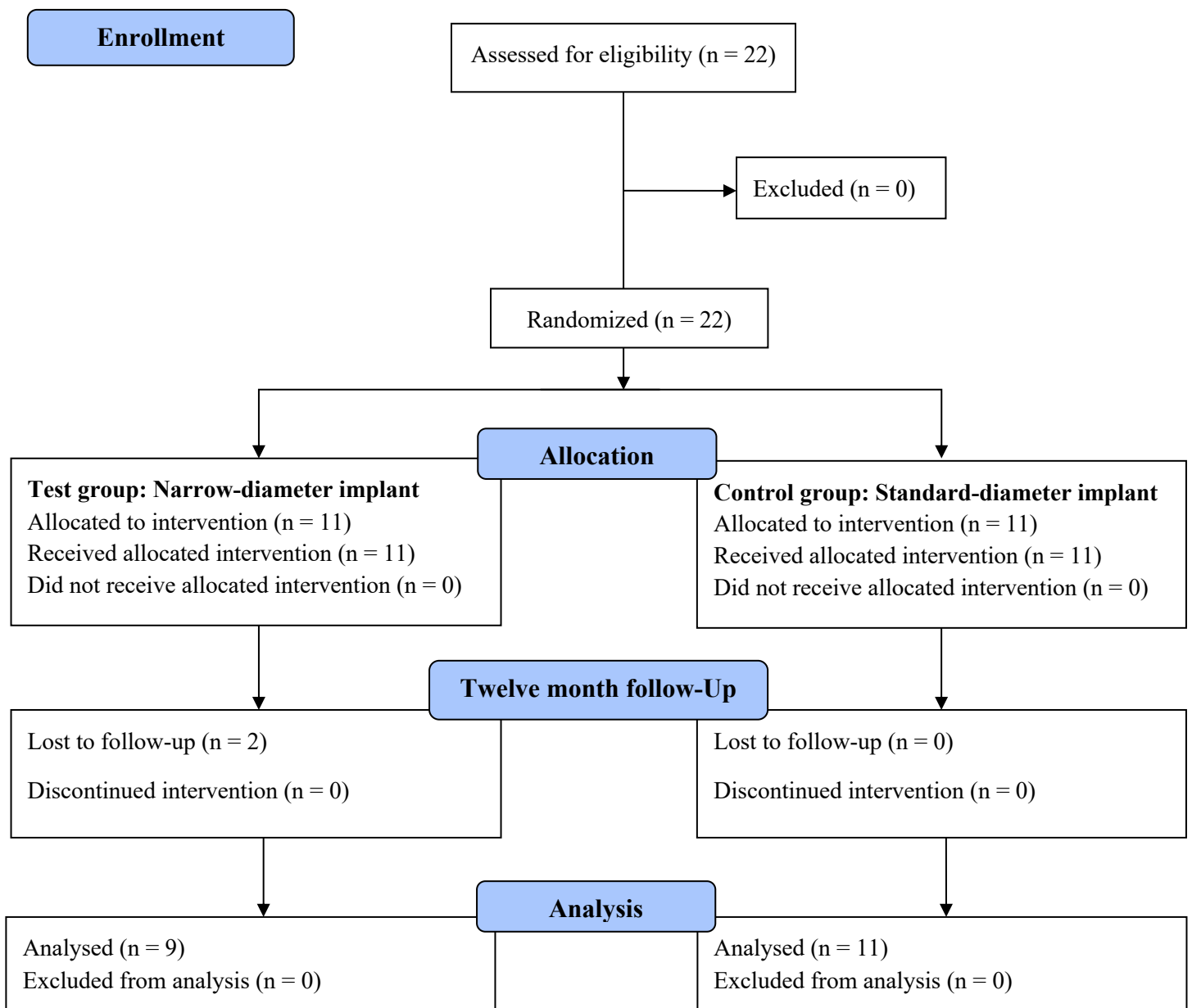

Supplement: Supplementary file 2 [file mmc2.pdf]
