# Supplementary material for: Narrow Versus Standard Diameter Implants for Supporting Single Crown Restorations in the Posterior Jaw: A Randomised Controlled Trial
Source: Int Dent J. 2025 Jan 26;75(3):2071–83. doi: 10.1016/j.identj.2024.12.031 (PMC12142787; doi:10.1016/j.identj.2024.12.031)
Supplement: Supplementary file 3 [file mmc3.docx]

**Table S1:** Characteristics of participants and implant sites

| Participants | Narrow diameter TiZr implants  (n = 11) | Standard diameter TiZr implants  (n = 11) | *P*-value |
| --- | --- | --- | --- |
| Gender (n)^1^  Female  Male | 7  4 | 3  8 | 0.09 |
| Age (years)^2^  Mean (SD) | 40.3 (9.2) | 47.5 (7.8) | 0.06 |
| ASA physical status^1^  ASA I  ASA II | 9  2 | 8  3 | 0.61 |
| Smoking habits (n)^1^  Non-smokers  Smokers | 10  1 | 10  1 | 1.00 |
| Implant sites | Narrow diameter TiZr implants  (n = 11) | Standard diameter TiZr implants  (n = 11) | *P*-value |
| Implant location (n)^1^  Mandible  Maxilla | 4  7 | 6  5 | 0.39 |
| Implant location (n)^1^  Premolars  Molars | 6  5 | 3  8 | 0.19 |
| Need for augmentation at time of implant placement (n)^1^  Yes  No | 4  7 | 1  10 | 0.13 |

ASA: American Society of Anesthesiologists; TiZr: titanium-zirconium; SD: standard deviation

^1^Chi-square test

^2^Independent *t*-test

**Table S2:** Peri-implant parameters (at one-year follow-up)

| Peri-implant parameters | Narrow diameter TiZr implants  (n = 9) | Standard diameter TiZr implants  (n = 11) | *P*-value |
| --- | --- | --- | --- |
| BoP (n)^1^  Yes  No | 2  7 | 1  10 | 0.41 |
| PPDs > 5 mm^1^  Yes  No | 1  8 | 0  11 | 0.26 |
| Width of KT < 2 mm^1^  Yes  No | 2  7 | 0  11 | 0.09 |

TiZr: titanium-zirconium; BoP: bleeding on probing; PPD: probing pocket depths; KT: keratinized tissue

^1^Chi-square test

**Table S3:** Esthetic outcomes

| Differences in esthetic parameters between the two groups at baseline | | | | |
| --- | --- | --- | --- | --- |
|  | Narrow diameter TiZr implant group^1^  mean (SD) | Standard diameter TiZr implant group^1^  mean (SD) | Mean difference and 95% CI | *P*-value |
| Mesial papilla | 1.36 (0.51) | 1.18 (0.41) | 0.18 (-0.23, 0.59) | 0.36 |
| Distal papilla | 1.18 (0.41) | 1.64 (0.67) | -0.46 (-0.95, 0.04) | 0.07 |
| Level of soft tissue margin | 1.27 (0.65) | 1.64 (0.51) | -0.36 (-0.88, 0.15) | 0.16 |
| Soft tissue contour | 1.36 (0.51) | 1.73 (0.47) | -0.36 (-0.80, 0.07) | 0.09 |
| Alveolar process | 1.36 (0.51) | 1.73 (0.47) | -0.36 (-0.80, 0.07) | 0.10 |
| Soft tissue color | 1.73 (0.47) | 1.82 (0.60) | -0.09 (-0.57, 0.39) | 0.70 |
| Soft tissue texture | 1.55 (0.52) | 1.82 (0.41) | -0.27 (-0.69, 0.14) | 0.19 |
| PES | 9.82 (2.23) | 11.55 (2.16) | -1.73 (-3.68, 0.23) | 0.08 |
| Differences in esthetic parameters between the two groups after one year | | | | |
|  | Narrow diameter TiZr implant group^1^  mean (SD) | Standard diameter TiZr implant group^1^  mean (SD) | Mean difference and 95% CI | *P*-value |
| Mesial papilla | 1.22 (0.67) | 1.45 (0.52) | -0.23 (-0.79, 0.33) | 0.39 |
| Distal papilla | 1.22 (0.44) | 1.64 (0.51) | -0.41 (-0.87, 0.04) | 0.07 |
| Level of soft tissue margin | 1.11 (0.33) | 1.27 (0.47) | -0.16 (-0.55, 0.23) | 0.40 |
| Soft tissue contour | 1.11 (0.33) | 1.27 (0.47) | -0.16 (-0.55, 0.23) | 0.40 |
| Alveolar process | 1.11 (0.33) | 1.45 (0.52) | -0.34 (-0.75, 0.06) | 0.09 |
| Soft tissue color | 1.44 (0.53) | 1.73 (0.65) | -0.28 (-0.85, 0.28) | 0.31 |
| Soft tissue texture | 1.44 (0.53) | 1.82 (0.41) | -0.37 (-0.83, 0.08) | 0.10 |
| PES | 8.67 (1.50) | 10.64 (2.46) | -1.97 (-3.94, 0.003) | 0.05 |

TiZr: titanium-zirconium; PES: pink esthetic score; CI: confidence interval; SD: standard deviation

^1^Independent *t*-test

**Table S4:** Patient-reported outcome measures in the first postoperative week (VAS 1-10)

|  | Narrow diameter TiZr implant group  mean (SD) | Standard diameter TiZr implant group  mean (SD) | Mean difference and 95% CI | *P*-value |
| --- | --- | --- | --- | --- |
| Day 1^1^  Pain  Swelling  Bleeding  Bruising  Root sensitivity of adjacent teeth | 3.82 (0.98)  3.55 (0.69)  3.18 (0.41)  2.45 (0.52)  2.36 (0.51) | 3.64 (1.12)  3.55 (1.13)  2.91 (0.54)  2.55 (0.52)  2.00 (0.63) | 0.18 (-0.76, 1.12)  0.00 (-0.83, 0.83)  0.27 (-0.15, 0.70)  -0.91 (-0.56, 0.37)  0.36 (-0.15, 0.87) | 0.69  1.00  0.20  0.69  0.15 |
| Day 2^1^  Pain  Swelling  Bleeding  Bruising  Root sensitivity of adjacent teeth | 3.91 (0.94)  3.55 (0.69)  2.55 (1.04)  1.45 (1.29)  1.64 (0.67) | 3.55 (0.93)  3.45 (1.04)  2.18 (0.98)  1.00 (1.18)  1.45 (0.52) | 0.36 (-0.47, 1.20)  0.09 (-0.69, 0.87)  0.36 (-0.53, 1.26)  0.46 (-0.65, 1.56)  0.18 (-0.36, 0.72) | 0.38  0.81  0.41  0.40  0.49 |
| Day 6^1^  Pain  Swelling  Bleeding  Bruising  Root sensitivity of adjacent teeth | 1.45 (0.82)  0.82 (0.98)  0.64 (0.92)  0.64 (0.92)  0.27 (0.47) | 1.55 (0.69)  1.27 (0.91)  0.82 (0.60)  0.55 (0.52)  0.45 (0.52) | -0.09 (-0.76, 0.58)  -0.46 (-1.29, 0.39)  -0.18 (-0.88, 0.52)  0.09 (-0.59, 0.77)  -0.18 (-0.62, 0.26) | 0.78  0.27  0.59  0.78  0.40 |

CI: confidence interval; TiZr: titanium-zirconium; SD: standard deviation; VAS: visual analogue scale

^1^Independent *t*-test
